# Towards quantifying uncertainty in predictions of Amazon ‘dieback’

**DOI:** 10.1098/rstb.2007.0028

**Published:** 2008-02-11

**Authors:** Chris Huntingford, Rosie A Fisher, Lina Mercado, Ben B.B Booth, Stephen Sitch, Phil P Harris, Peter M Cox, Chris D Jones, Richard A Betts, Yadvinder Malhi, Glen R Harris, Mat Collins, Paul Moorcroft

**Affiliations:** 1 Centre for Ecology and HydrologyBenson Lane, Wallingford, Oxon OX10 8BB, UK; 2 Department of Animal and Plant Sciences, University of SheffieldWestern Bank, Sheffield S10 2TN, UK; 3 Met Office Hadley CentreFitzRoy Road, Exeter, Devon EX1 3PB, UK; 4 Met Office Hadley Centre, JCHMRBenson Lane, Wallingford, Oxon OX10 8BB, UK; 5 School of Engineering, Computer Science and MathematicsHarrison Building, North Park Road, University of Exeter, Exeter EX4 4QF, UK; 6 Oxford University Centre for the Environment, University of OxfordSouth Parks Road, Oxford OX1 3QY, UK; 7 Department of Organismic and Evolutionary Biology, Harvard UniversityHUH, 22 Divinity Avenue, Cambridge, MA 02138, USA

**Keywords:** climate change, carbon cycle, global warming, Amazonia, die-back, ecosystems

## Abstract

Simulations with the Hadley Centre general circulation model (HadCM3), including carbon cycle model and forced by a ‘business-as-usual’ emissions scenario, predict a rapid loss of Amazonian rainforest from the middle of this century onwards. The robustness of this projection to both uncertainty in physical climate drivers and the formulation of the land surface scheme is investigated. We analyse how the modelled vegetation cover in Amazonia responds to (i) uncertainty in the parameters specified in the atmosphere component of HadCM3 and their associated influence on predicted surface climate. We then enhance the land surface description and (ii) implement a multilayer canopy light interception model and compare with the simple ‘big-leaf’ approach used in the original simulations. Finally, (iii) we investigate the effect of changing the method of simulating vegetation dynamics from an area-based model (TRIFFID) to a more complex size- and age-structured approximation of an individual-based model (ecosystem demography).

We find that the loss of Amazonian rainforest is robust across the climate uncertainty explored by perturbed physics simulations covering a wide range of global climate sensitivity. The introduction of the refined light interception model leads to an increase in simulated gross plant carbon uptake for the present day, but, with altered respiration, the net effect is a decrease in net primary productivity. However, this does not significantly affect the carbon loss from vegetation and soil as a consequence of future simulated depletion in soil moisture; the Amazon forest is still lost. The introduction of the more sophisticated dynamic vegetation model reduces but does not halt the rate of forest dieback. The potential for human-induced climate change to trigger the loss of Amazon rainforest appears robust within the context of the uncertainties explored in this paper. Some further uncertainties should be explored, particularly with respect to the representation of rooting depth.

## 1. Introduction

When forced by a ‘business-as-usual’ (IS92a) emissions scenario, a version of the Hadley Centre general circulation model (GCM) (Gordon *et al*. 2000) extended to model the global carbon cycle (including a dynamic global vegetation model (DGVM)) predicts that climate change could cause a major loss of the Amazon rainforest (Cox *et al*. 2000). Besides acting as a positive feedback on climate, whereby additional carbon dioxide is released back into the atmosphere, the loss of the rainforest in itself would clearly be a significant environmental matter. Cox *et al*. (2004) suggest that the main driver of such ‘dieback’ could, qualitatively, be related to GCM projections of persistent ‘El Niño-like’ oceanic conditions, triggering major rainfall reductions over the Amazon Basin. Further analysis by Harris *et al*. (2008) demonstrates that the changes in rainfall may be more complex and that the modelled GCM drying is also forced by predicted changes in the gradient of Atlantic sea surface temperatures between the Northern and Southern Hemispheres. Cox *et al*. (in preparation) analyse the Amazonian drought of 2005, and provide evidence that, for that particular year, the north–south gradient of Atlantic sea surface temperatures was anomalously high. The particular format and timing of the drought are consistent with modelled emerging signals by HadCM3.

It is important to understand the uncertainties related to the Cox *et al*. (2000) prediction of dieback. These can arise through uncertainty in both simulated regional climate change and the modelled land surface response. We use perturbed physics ensembles of HadCM3 (part of the ‘quantifying uncertainty in model predictions’ (QUMPs) initiative) to explore uncertainties in the predicted climate drivers affecting future Amazon rainforest stability. The ensembles extend the Murphy *et al*. (2004) work to fully transient simulations of historical and future climate (using the SRES A1B scenario; Nakićenović & Swart 2000; Collins *et al*. 2006; Murphy *et al*. 2007). Two uncertainties in the structure of the land surface model are considered, where enhanced ecological realism addresses potential weaknesses in the original modelling system. First, we introduce a more advanced representation of canopy light interception containing an explicit description of interception for different canopy levels (Sellers 1985), giving a multilayer approach to scaling from leaf- to canopy-level photosynthesis. Second, we consider the contribution of the representation of vegetation dynamics to the dieback response, by replacing the TRIFFID DGVM with the ecosystem demography (ED) model (Moorcroft *et al*. 2001). ED is a size- and age-structured approximation of an individual-based gap model (Friend *et al*. 1997), modified to allow the gap model vegetation dynamics to be employed at large spatial scales. The ED individual-based model with its enhanced biophysical representation of vegetation is a logical step on from the TRIFFID model; the latter has a more empirical representation of vegetation competition and interaction. As both models are driven with the same canopy photosynthesis and surface exchange scheme, the contribution of modelled vegetation dynamics to dieback is isolated from that of plant physiology.

## 2. Analysis

GCM simulations of century-scale climate change typically take three months to complete, even with supercomputing facilities. They are highly sophisticated numerical models of the climate, but these two aspects make it difficult to explore new numerical depictions of Earth system processes, such as the land surface response. Hence, a spectrum of modelling tools is required where complexity is retained in the processes of interest, but other components of the Earth system are approximated. The Integrated Model Of Global Effects of climatic aNomalies model (IMOGEN) strives to achieve this, combining the ‘GCM analogue model’ to emulate surface climate (Huntingford & Cox 2000) but with the full GCM land surface model. IMOGEN is described in Huntingford *et al*. (2004), where it was applied to the early analysis of potential Amazonian dieback.

In the standard IMOGEN system, CO_2_ emissions are prescribed and the model simulates terrestrial carbon fluxes from the land surface scheme and oceanic fluxes using the impulse response function of Joos *et al*. (1996), generating atmospheric CO_2_ concentrations. Here, we analyse ED model projections for the Amazon Basin, noting that ED was originally developed with plant functional types (PFTs) specific to Amazonia (Moorcroft *et al*. 2001). ED is not yet fully tested for temperate and boreal regions, preventing predictions of the global land–atmosphere net carbon exchange and hence atmospheric CO_2_ content for prescribed emissions of CO_2_. Instead, IMOGEN is run with prescribed CO_2_ concentrations identical to those derived by the Hadley Centre GCM contribution to the Coupled Carbon Cycle Climate Model Intercomparison Project (C_4_MIP; Friedlingstein *et al*. 2006). Although the ability of the land surface to affect atmospheric CO_2_ concentrations through large-scale biogeochemical feedbacks is lost, the vegetation change in Amazonia was responsible for only 10% of the total biosphere–atmosphere positive feedback predicted by Cox *et al*. (2000, 2004).

Huntingford & Cox (2000) demonstrate that, to a reasonable level of accuracy, surface climate (by both geographical position and season) as depicted by HadCM3 transient simulations exhibits linearity in global mean temperature over land. We recalculate such propagating patterns based on each member of the QUMP perturbed physics ensemble, and hence the IMOGEN system explores how Amazon dieback is sensitive to different predictions of surface climate. The IMOGEN system is also used to consider how altered representations of light interception and vegetation dynamics influence the rainforest response to simulated drying and raised temperatures.

For the IMOGEN simulations we perform, the trajectory of climatic forcing is similar to that of the QUMP simulations themselves (so the GCM ‘analogue model’ component of IMOGEN could have been overridden with direct climatological predictions from the QUMP ensemble). However, the existence of propagating patterns of climatological change allows extrapolation of existing GCM simulations to a range of different emission profiles. Hence, the system presented below is now available for future simulations corresponding to a diverse range of future pathways in atmospheric greenhouse gas concentrations, including uncertainty bounds based on the QUMP simulations.

### (a) Perturbed physics simulations

Climatological driving data required by IMOGEN are created based on 16 perturbed physics transient HadCM3 simulations. These simulations translate uncertainties first explored in Murphy *et al*. (2004) and extended by Webb *et al*. (2006) into transient climate responses over the historical period and future (to the year 2100, using the SRES A1B scenario) by incorporating a dynamical ocean component. The 16-member ensemble samples uncertainties in cloud and atmospheric processes, land surface and sea ice parametrizations. The methodology for these simulations is described in Collins *et al*. (2006), although our analysis uses a subsequently refined set of 16 perturbed physics simulations with reduced biases in sea temperatures in the Atlantic and Arctic oceans (Murphy *et al*. 2007). A limitation of this approach is that the model uncertainty is explored within a single model framework and major structural perturbations to the model physics are not sampled. However, a comparison between this ensemble and the multi-model ensemble predictions used by the Intergovernmental Panel on Climate Change (IPCC) Fourth Assessment shows a similar spread in the regional surface temperature response (Meehl *et al*. 2007, fig. 10.30), suggesting that the QUMP ensemble explores a comparable range of uncertainty. Murphy *et al*. (2007) present an extended discussion on the limitations and merits of both the perturbed physics and multi-model ensemble approaches for quantifying modelling uncertainty.

A pre-industrial climatology is derived from the control state from each of the perturbed physics simulations, corresponding to a ‘perpetual 1860s climate’. Monthly mean changes in near-surface climate variables are derived for each gridbox from the transient simulations, in an identical way to that of Huntingford & Cox (2000), thereby giving the analogue model components for the ensemble of IMOGEN simulations. IMOGEN calculates the surface fluxes of heat, vapour and carbon dioxide using v. 2.2 of the Met Office Surface Exchange Scheme (Moses; Cox *et al*. 1998, 1999 for Moses v. 2.1, and extension to v. 2.2, Essery *et al*. 2003), coupled to the TRIFFID DGVM (Cox 2001), hence generating a land surface model similar to that of the Cox *et al*. (2000) GCM simulation. A caveat is that the new IMOGEN simulations based on the perturbed physics ensembles do not include the influence of dieback on regional surface climate; such local biophysical feedbacks can be important (see Gash & Nobre 1997; Nepstad *et al*. 1999; Betts *et al*. 2004). Other potential biogeochemical feedbacks (which are not yet in GCMs either) such as changes in emissions of dust (in the event of a complete loss of vegetation) and isoprenes are also neglected (Sanderson *et al*. 2003; Woodward *et al*. 2005; Betts *et al*. 2008).

**Figure 1:**
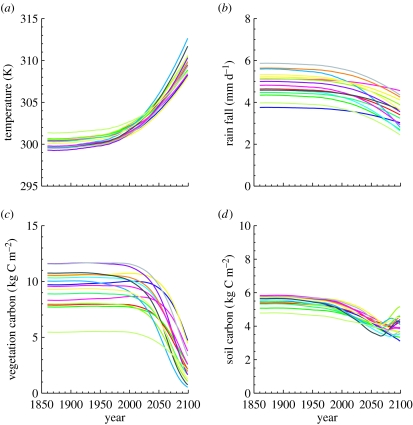
Changes in (*a*) mean temperature and (*b*) rainfall for the Amazon region (see fig. 1 of Huntingford *et al*. (2004) for precise region) from a perturbed physics ensemble of HadCM3 forced with historical and SRES A1B changes in greenhouse gases and other forcing agents. The changes in both (*c*) vegetation and (*d*) soil carbon for the Amazon region are derived from the IMOGEN modelling system.

Projections of change in temperature and rainfall for the Amazon Basin by the perturbed physics ensemble of transient simulations are presented in figure 1*a*,*b*. Calculated influence on vegetation and soil carbon using the IMOGEN modelling system is also shown in figure 1*c*,*d*. Although the initial vegetation and soil carbon states differ for the 16 perturbed physics climatologies, the temporal dynamic is robust. The TRIFFID DGVM driven with all the 16 climatologies simulates large-scale forest dieback across Amazonia, predominantly associated with drought-induced reductions in plant productivity. There are also major reductions in soil carbon (the slight reversal in some simulations of soil carbon content is where dieback is sufficiently fast that litter input to the soil ‘overtakes’ increased soil respiration losses due to raised temperatures). This suggests that predicted Amazonian dieback is robust to multiple parameter perturbations in HadCM3.

### (b) Impact of a multilayer canopy light interception model

The Moses land surface scheme used by Cox *et al*. (2000) assumes the functioning of the plant canopy scales as a ‘big-leaf’ and follows Beer's law. We introduce a more realistic depiction of light levels within a canopy (Jogireddy *et al*. 2006; Mercado *et al*. 2007), calculate its effect on stomatal conductance and thus control on photosynthesis and evaporation, and determine the impact on modelled vegetation and soil carbon for the Amazon rainforest during the twenty-first century. We include an explicit scaling-up from leaf-to-canopy, using a multilayer canopy radiation interception algorithm based on an analytical two-stream model (Sellers 1985). For such a multilayer approach, absorption and scattering losses of incident radiation, for both direct and diffuse radiation, are calculated at different levels in the canopy. These include contributions from the visible and near-infrared wavebands, from which the absorbed photosynthetically active radiation (PAR) is derived. Using the calculated absorbed PAR at each layer of the canopy, leaf photosynthesis, leaf respiration and stomatal conductance are calculated and summed to provide canopy values. The parametrization of the vertical profile of leaf nitrogen through the canopy has also been modified to follow observations from a site in central Amazonia (Mercado *et al*. 2007). The observed vertical profile of nitrogen is less steep than that predicted under the original Beer's law (this implies higher total canopy nitrogen when observed profiles are used). The improved light interception model has been successfully tested against eddy correlation measurements for a rainforest site in Manaus (Mercado *et al*. 2007); there the authors found the main improvement of introducing the ‘two-stream/multilayer’ description was to allow a more realistic modelling of the response of photosynthesis to light, and the associated impact on the diurnal cycle of modelled carbon and water fluxes. The Moses-modelled photosynthesis tends to saturate quickly for increasing solar radiation, generating a ‘flat’ response in the middle of the day, whereas measurements indicate photosynthetic response to varying light levels for the entire diurnal period.

**Figure 2:**
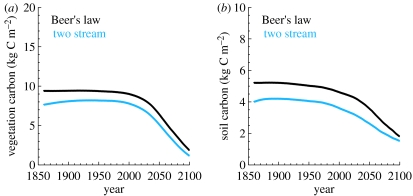
Changes in Amazonian (*a*) vegetation and (*b*) soil carbon (for the same region as used in figure 1) using an IMOGEN initial climatology and climate change patterns derived from the original Cox *et al*. (2000) simulation. The black curve corresponds to the standard ‘big-leaf’ version of the land surface scheme and the blue curve the review ‘two-stream’ approach to light interception.

The introduced scheme simulates higher gross primary production, but lower net primary production (and thus lower plant and soil carbon pools relative to the original big-leaf simulation; figure 2). This is as a consequence of significantly higher plant respiration costs associated with the higher canopy nitrogen contents. Overall, this improved treatment of radiation absorption yields little alteration (when using prescribed patterns of climate change based on the HadCM3 simulation of Cox *et al*. (2000)) to the original dieback result obtained with the standard Moses model (figure 2). The dominant cause of dieback remains to be the prescribed reduced rainfall causing severe soil moisture stress, affecting both simulations (figure 2) independent of the improved description of photosynthetic behaviour.

### (c) Introduction of the ED model

Cox *et al*. (2000) used the TRIFFID DGVM, based on a large-scale competition between trees, shrubs and grasses. The dominant cover is determined by the balance between ability to ‘fix’ carbon by photosynthesis and loss of carbon by litterfall. The combination of both modelled warming and simultaneous rainfall decreases by HadCM3 means that trees are projected to become unsustainable, and the dominant vegetation type then becomes shrubs. Towards the end of the twenty-first century, these are superseded by first grasses and finally desert. The TRIFFID model is described in Cox (2001) and the behaviour of the dominant vegetation class and route to dieback is given in Huntingford *et al*. (2000). We replaced the TRIFFID DGVM with the ED model in IMOGEN. The ED model (Moorcroft *et al*. 2001) is unique among DGVMs using a size- and age-structured approximation of a gap model, to allow both operation at a large spatial scale and representation of vegetation dynamics, turnover, competition and mortality in an ecologically realistic fashion. ED is controlled by parameters that are more amenable to ground measurements (a criticism of existing DGVMs is that their parametrization of vegetation dynamics, competition and species replacement is often difficult to constrain with ecological data). The parameters of the vegetation dynamics component (including specific leaf area, wood density, leaf lifespan, mortality rates, allocation patterns and PFTs) were all derived from ground measurements by Moorcroft *et al*. (2001), and with an emphasis on the Amazon region. The model was verified against vegetation composition data, showing that ED can successfully predict vegetation growth, composition and spatial distribution under contrasting conditions within the Amazon region.

In Moses–TRIFFID, each sub-grid cell tile contains a single PFT (there are five possible PFTs; also possible are ice, lake, urban and soil/desert land uses). ED, in contrast, defines the sub-grid cell tiles according to a model of ecosystem disturbance (caused by either mortality or fire). Each tile consists of land area which has a common ‘age since last disturbance’. Each tile is populated with multiple ‘cohorts’ of trees, which represent groups of individuals with a common PFT and height class. Cohorts of different PFTs may coexist vertically within each tile. Competition for light between different PFTs is simulated, a development which greatly enhances the process representation of vegetation competition and dynamics compared with other existing global vegetation models. The age-structured model represents heterogeneity in the light environment within a grid cell, and therefore it simulates the dominance of fast-growing species with high-mortality rates in high-light environments that have recently been disturbed, and their successional replacement by slow-growing low-mortality species through time. The model can therefore estimate the regrowth of biomass and leaf area after disturbance events such as mortality. The ED model used in this analysis uses five PFTs similar to that used in Moorcroft *et al*. (2001); that is, C3 and C4 grasses, plus three types of broadleaf tree: ‘early successional’; ‘mid-successional’; and ‘late successional’.

The original Moses gas exchange and photosynthesis model was retained (Essery *et al*. 2003), but updated to allow multiple canopy layers. The sub-grid cell tiling structure of the vegetated land surface was changed to the age class-defined tiles used in the ED model. ED has a monthly time step and provides the tiling structure plus the height, PFT and leaf area index of each cohort to the Moses gas exchange model, which in turn calculates net primary productivity (NPP) for each cohort. These derived hourly values of NPP are integrated and passed back to the ED model after each modelled month to update vegetation growth and mortality.

**Figure 3:**
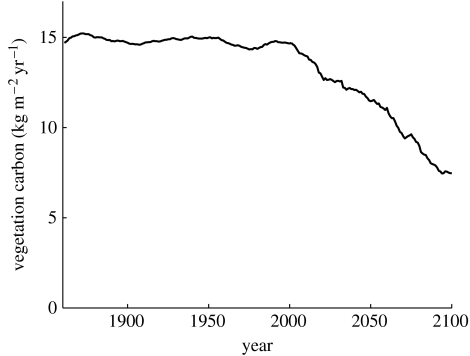
Change in vegetation carbon predicted by the ED model (as placed in the IMOGEN modelling structure) for Amazonia (the same region as used in figure 1). The simulation is for a control climate based on the CRU climatology, with climate change anomalies based on HadCM3.

**Figure 4:**
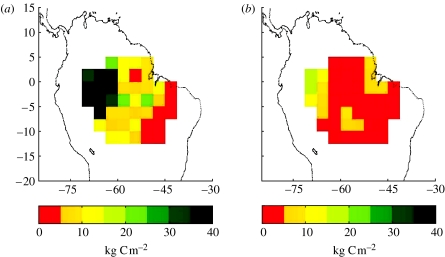
Spatial representation of vegetation carbon for the identical simulation with the ED model as given in figure 3. (*a*) The pre-industrial period and (*b*) centred on the last decade of the twenty-first century.

Figure 3 shows Amazon vegetation carbon simulated by ED and forced with IMOGEN climate patterns based on the HadCM3 simulation of Cox *et al*. (2000). ED was unable to simulate forest until the initial climatology (representative of pre-industrial times) was replaced with the one based on the Climatic Research Unit (CRU) climatology (New *et al*. 2000). It is known that HadCM3 has a slight dry bias in its control climate for Amazonia to which ED was responding. The ED model simulates future reductions in Amazonian vegetation in the twenty-first century in response to climate change modelled by HadCM3. However, the rate of dieback is significantly slower than with TRIFFID, indicating possibly a greater forest resilience. Spatial changes in Amazon terrestrial carbon are given in figure 4.

## 3. Discussion and conclusions

The impact of uncertainties in modelled climate response on Amazon rainforest sustainability for increasing concentrations of atmospheric greenhouse gases has been investigated using a ‘perturbed physics ensemble’. In the context of predictions by other modelling centres, the HadCM3 ensemble spans the range of global mean temperature responses in the AR4 multi-model ensemble (Collins *et al*. 2006), but samples a smaller range of the precipitation uncertainty in the Amazon region. Cox *et al*. (2004) suggest a relationship between wet season precipitation and trends in the future El Niño-Southern Oscillation (ENSO) state. The ensemble members share to a greater or lesser extent the tendency in the original HadCM3 response towards an enhanced El Niño-like state in the future (and hence wet season reduction of rainfall). However, Collins *et al*. (2005) conclude that across different GCMs, there is a roughly equal likelihood between El Niño or La Niña trends among the multi-model ensemble. Good *et al*. (2008) illustrate further a linkage between shifts in the Intertropical Convergence zone and the dry season rainfall in this region. That the perturbed physics ensemble does not capture the full range in future rainfall responses is an important caveat that should be addressed in future work. Nevertheless, the response of climate drivers in the HadCM3 family of models remains credible for the Amazon region and the robustness of the dieback result to the uncertainty in climate drivers for that GCM represents a substantial step forward in predictions. IMOGEN, now calibrated against the QUMP ensemble, is available to assess the likelihood of dieback for a range of emissions trajectories. These could include pathways to atmospheric stabilization (e.g. those of Wigley *et al*. 1996) or the emerging concept of climate ‘overshoot’ (e.g. Huntingford & Lowe 2007), whereby a potentially dangerous level of climate change is found to have been passed, followed by massive reductions in emissions in an attempt to fall back below that level. For the Amazon rainforest, this raises issues regarding hysteresis and recovery from any dieback.

The sensitivity of modelled Amazon dieback to the description of the land surface model has been explored. In parallel calculations (see Sitch *et al*. in press), five DGVMs are coupled to IMOGEN (again with patterns of climate change based on HadCM3) and forced with four different SRES CO_2_ emission scenarios. The quantitative response of the DGVMs to drought differs among models, with TRIFFID and Hyland DGVMs most sensitive to reduced rainfall and elevated temperatures across Amazonia, whereas LPJ and Orchidee simulate moderate forest dieback. Salazar *et al*. (2007) run the CPTEC-PVM (Oyama & Nobre 2004) potential vegetation model with future climatologies from 15 climate models and for two different SRES emission scenarios (A2 and B1). Their results project a reduction in forest coverage for all simulations despite large uncertainties in both magnitude and sign of climate model projections of future rainfall across Amazonia. Salazar *et al*. (2007) highlight that for GCMs predicting higher future rainfall amounts across the Amazon Basin, elevated temperatures alone are sufficient to cause conversion of forest to savannah ecosystems. Hence, Amazon rainforest dieback may be less sensitive to the choice of GCM pattern of changing rainfall than hitherto expected. We have targeted two particular aspects of Amazon's response: first, the introduction of a multilayer two-stream canopy light module, thus improving the light response of photosynthesis and diurnal cycles of carbon and water fluxes, and, second, the adoption of ED, a DGVM that represents a first step towards incorporating a greater process-based understanding of vegetation dynamics, turnover, competition and mortality. In all circumstances, we find that dieback is still probable by the end of the twenty-first century for the business-as-usual emission profile selected.

Deforestation has a large impact on tropical forests (Achard *et al*. 2002). The effects of both deforestation and global warming are predicted to negatively impact Amazonian forest extent (Cramer *et al*. 2004; Salazar *et al*. 2007) and change both regional and global climate (Sitch *et al*. 2005; Costa *et al*. 2007). To further improve our ability to project the fate of Amazonian forests, ecosystem models need to incorporate land use and cover changes.

Amazonian ecosystem models need further verification against carbon and water flux data. The majority of flux tower and experimental studies in the region do not detect any hydraulic limitation of evapotranspiration or gross primary productivity in the dry season, with many attributing this behaviour to the existence of deep roots (Hodnett *et al*. 1995; Grace *et al*. 1996; Araújo *et al*. 2002; Carswell *et al*. 2002; Saleska *et al*. 2003; da Rocha *et al*. 2004; Goulden *et al*. 2004; Fisher *et al*. 2007; Nepstad *et al*. 2007). Two examples where hydraulic limitation was measured are Malhi *et al*. (1998; which was tested against the IMOGEN surface model by Harris *et al*. (2004)) and a more recent manipulation study (Fisher *et al*. 2007) finding that when a 50% reduction in through-fall was imposed on the forest, a large (up to 80%) reduction in forest transpiration (by implication, photosynthesis) resulted within a single year. These results suggest that the deep roots do not entirely buffer the forest from the imposed dry conditions and comparison of model predictions against all these observations remains a high research priority. It is probable that alterations of modelling rooting depths and the responses of vegetation to high temperatures (Salazar *et al*. 2007) are necessary to correctly simulate contemporary and future patterns of gas exchange. If the total rainfall falls below a threshold defined by the total evaporative demand, the effect of rainfall storage in the dry season becomes unimportant, so the impact of deep roots will probably delay the impact of any drying and dieback, but not be able to prevent it entirely if this threshold is breached.

We have shown that the dieback result of Cox *et al*. (2000) is robust within the structural constraints of HadCM3 climatology across the existing atmosphere parameter uncertainty. Large-scale forest dieback across Amazonia is a robust projection with enhanced representations of canopy light interception and with a more process-based DGVM, ED. The ED model was parametrized independently of any GCM, hence eliminating the risk of compensating biases between the climate and land surface models. These results, taken together with findings from other recent studies using multiple DGVMs, climate models and projections of land use and cover change, suggest that the Amazon rainforest must be considered to be highly vulnerable to future global change induced by raised concentrations of atmospheric greenhouse gases.

## References

[bib1] Achard F, Eva H.D, Stibig H.-J, Mayaux P, Gallego J, Richards T, Malingreau J.P (2002). Determination of deforestation rates of the world's humid tropical forests. Science.

[bib2] Araújo A.C (2002). Comparative measurements of carbon dioxide fluxes from two nearby towers in a central Amazonian rainforest: the Manaus LBA site. J. Geophys. Res. Atmos.

[bib3] Betts R.A, Cox P.M, Collins M, Harris P.P, Huntingford C, Jones C.D (2004). The role of ecosystem–atmosphere interactions in simulated Amazonian precipitation decrease and forest die-back under global climate warming. Theor. Appl. Climatol.

[bib4] Betts R, Sanderson M, Woodward S (2008). Effects of large-scale Amazon forest degradation on climate and air quality through fluxes of carbon dioxide, water, energy, mineral dust and isoprene. Phil. Trans. R. Soc. B.

[bib5] Carswell F.E (2002). Seasonality in CO_2_ and H_2_O flux at an eastern Amazonian rain forest. J. Geophys. Res. Atmos.

[bib6] Collins M (2005). El Niño- or La Niña-like climate change?. Clim. Dynam.

[bib7] Collins M, Booth B.B.B, Harris G.R, Murphy J.M, Sexton D.M.H, Webb M.J (2006). Towards quantifying uncertainty in transient climate change. Clim. Dynam.

[bib8] Costa M.H, Yanagi S.N.M, Souza P.J.O.P, Ribeiro A, Rocha E.J.P (2007). Climate change in Amazonia caused by soybean cropland expansion, as compared to caused by pastureland expansion. Geophys. Res. Lett.

[bib9] Cox, P. M. 2001 Description of the TRIFFID dynamic global vegetation model. Technical note 24, Hadley Centre, Met Office, Exeter, UK.

[bib14] Cox P.M, Huntingford C, Harding R.J (1998). A canopy conductance and photosynthesis model for use in a GCM land surface scheme. J. Hydrol.

[bib10] Cox P.M, Betts R.A, Bunton C, Essery R.L.H, Rowntree P.R, Smith J (1999). The impact of new land surface physics on the GCM simulation of climate and climate sensitivity. Clim. Dynam.

[bib12] Cox P.M, Betts R.A, Jones C.D, Spall S.A, Totterdell I.J (2000). Acceleration of global warming due to carbon-cycle feedbacks in a coupled climate model. Nature.

[bib11] Cox P.M, Betts R.A, Collins M, Harris P.P, Huntingford C, Jones C.D (2004). Amazonian forest die-back under climate-carbon cycle projections for the 21st century. Theor. Appl. Climatol.

[bib13] Cox, P. M., Harris, P. P., Huntingford, C., Betts, R. A., Collins, M., Jones, C. D., Marengo, J. & Nobre, C. In preparation. The 2005 Amazonian drought in the context of climate change.

[bib16] Cramer W, Bondeau A, Schaphoff S, Lucht W, Smith B, Sitch S (2004). Tropical forests and the global carbon cycle: impacts of atmospheric carbon dioxide, climate change and rate of deforestation. Phil. Trans. R. Soc. B.

[bib17] da Rocha H.R, Goulden M.L, Miller S.D, Menton M.C, Pinto L.D.V.O, de Freitas H.C, Figueira A.M.E.S (2004). Seasonality of water and heat fluxes over a tropical forest in eastern Amazonia. Ecol. Appl.

[bib18] Essery R.L.H, Best M.J, Betts R.A, Cox P.M, Taylor C.M (2003). Explicit representation of subgrid heterogeneity in a GCM land surface scheme. J. Hydrometeorol.

[bib19] Fisher R.A, Williams M, Lola da Costa A, Malhi Y, da Costa R.F, Almeida S, Meir P (2007). The response of an Eastern Amazonian rain forest to drought stress: results and modelling analyses from a throughfall exclusion experiment. Glob. Change Biol.

[bib20] Friedlingstein P (2006). Climate-carbon cycle feedback analysis: results from the (CMIP)-M-4 model intercomparison. J. Clim.

[bib21a] Friend A.D, Stevens A.K, Knox R.G, Cannell M.G.R (1997). A process-based, terrestrial biosphere model of ecosystem dynamics (Hybrid v3.0). Ecol. Model.

[bib21b] Gash J.H.C, Nobre C.A (1997). Climatic effects of Amazonian deforestation: some results from ABRACOS. Bull. Am. Meteorol. Soc.

[bib21] Good P, Lowe J.A, Collins M, Moufouma-Okia W (2008). An objective tropical Atlantic sea surface temperature gradient index for studies of south Amazon dry-season climate variability and change. Phil. Trans. R. Soc. B.

[bib22] Gordon C, Cooper C, Senior C.A, Banks H, Gregory J.M, Johns T.C, Mitchell J.F.B, Wood R.A (2000). The simulation of SST, sea ice extents and ocean heat transports in a version of the Hadley Centre coupled model without flux adjustments. Clim. Dynam.

[bib23] Goulden M.L, Miller S.D, da Rocha H.R, Menton M.C, de Freitas H.C, Figueira A.M.E.S, de Sousa C.A.D (2004). Diel and seasonal patterns of tropical forest CO_2_ exchange. Ecol. Appl.

[bib24] Grace J, Malhi Y, Lloyd J, McIntyre J, Miranda A.C, Meir P, Miranda H.S (1996). The use of eddy covariance to infer the net carbon dioxide uptake of Brazilian rain forest. Glob. Change Biol.

[bib25a] Harris P.P, Huntingford C, Gash J.H.C, Hodnett M.G, Cox P.M, Malhi Y, Araujo A.C (2004). Calibration of a land-surface model using data from primary forest sites in Amazonia. Theoret. Appl. Climatol.

[bib25] Harris P.P, Huntingford C, Cox P.M (2008). Amazon basin climate under global warming: the role of the sea-surface temperature. Phil. Trans. R. Soc. B.

[bib26a] Hodnett M.G, Dasilva L.P, Darocha H.R, Senna R.C (1995). Seasonal soil water storage changes beneath central Amazonian rainforest and pasture. J. Hydrol.

[bib26] Huntingford C, Cox P.M (2000). An analogue model to derive additional climate change scenarios from existing GCM simulations. Clim. Dynam.

[bib29] Huntingford C, Lowe J (2007). Overshoot scenarios and climate change. Science.

[bib27] Huntingford C, Cox P.M, Lenton T.M (2000). Contrasting responses of a simple terrestrial ecosystem model to global change. Ecol. Model.

[bib28] Huntingford C, Harris P.P, Gedney N, Cox P.M, Betts R.A, Marengo J.A, Gash J.H.C (2004). Using a GCM analogue model to investigate the potential for Amazonian forest die-back. Theor. Appl. Climatol.

[bib30] Jogireddy, V., Cox, P. M., Huntingford, C., Harding, R. J. & Mercado, L. M. 2006 An improved description of canopy light interception for use in a GCM land-surface scheme: calibration and testing against carbon fluxes at a coniferous forest. Hadley Centre Technical note 63, The Hadley Centre, Exeter, UK.

[bib31] Joos F, Bruno M, Fink R, Siegenthaler U, Stocker T.F (1996). An efficient and accurate representation of complex oceanic and biospheric models of anthropogenic carbon uptake. Tellus B.

[bib32a] Malhi Y, Nobre A.D, Grace J, Kruijt B, Pereira M.G.P, Culf A, Scott S (1998). Carbon dioxide transfer over a Central Amazonian rain forest. J. Geophys. Res.

[bib32] Meehl G.A, Solomon S, Qin D, Manning M, Chen Z, Marquis M, Averyt K.B, Tignor M, Miller H.L (2007). Global climate projections. Climate change 2007: the physical science basis. Contribution of working group I to the fourth assessment report of the Intergovernmental Panel on Climate Change.

[bib33] Mercado L.M, Huntingford C, Gash J.H.C, Cox P.M, Jogireddy V (2007). Improving the representation of radiation interception and photosynthesis for climate model applications. Tellus B.

[bib34] Moorcroft P.R, Hurtt G.C, Pacala S.W (2001). A method for scaling vegetation dynamics: the ecosystem demography model (ED). Ecol. Monogr.

[bib36] Murphy J.M, Sexton D.M.H, Barnett D.N, Jones G.S, Webb M.J, Collins M, Stainforth D.A (2004). Quantification of modelling uncertainties in a large ensemble of climate change simulations. Nature.

[bib35] Murphy J.M, Booth B.B.B, Collins M, Harris G, Sexton D, Webb M (2007). A methodology for probabilistic predictions of regional climate change from perturbed physics ensembles. Phil. Trans. R. Soc. A.

[bib37] Nakićenović N, Swart R (2000). IPCC special report on emissions scenarios.

[bib38] Nepstad D.C (1999). Large-scale impoverishment of Amazonian forests by logging and fire. Nature.

[bib40a] Nepstad D.C, Tohver I.M, Ray D, Moutinho P, Cardinot G (2007). Mortality of large trees and lianas following experimental drought in an amazon forest. Ecology.

[bib40] New M, Hulme M, Jones P (2000). Representing twentieth-century space–time climate variability. Part II: development of 1901–96 monthly grids of terrestrial surface climate. J. Clim.

[bib41] Oyama M.D, Nobre C.A (2004). A simple potential vegetation model for coupling with the simple biosphere model (SIB). Rev. Bras. Meteorol.

[bib43] Salazar L.F, Nobre C.A, Oyama M.D (2007). Climate change consequences on the biome distribution in tropical South America. Geophys. Res. Lett.

[bib44] Saleska S.R (2003). Carbon in Amazon forests: unexpected seasonal fluxes and disturbance-induced losses. Science.

[bib45] Sanderson M.G, Jones C.D, Collins W.J, Johnson C.E, Derwent R.G (2003). Effect of climate change on isoprene emissions and surface ozone levels. Geophys. Res. Lett.

[bib46] Sellers P.J (1985). Canopy reflectance, photosynthesis and transpiration. Int. J. Remote Sens.

[bib47] Sitch S, Brovkin V, von Bloh W, van Vuuren D, Ganopolski A (2005). Impacts of future land cover changes on atmospheric CO_2_ and climate. Glob. Biogeochem. Cycles.

[bib48] Sitch, S. *et al* In press. Evaluation of the terrestrial carbon cycle, future plant geography and climate-carbon feedbacks using 5 Dynamic Global Vegetation Models (DGVMs). *Glob. Change. Biol.*

[bib50a] Webb M.J (2006). On the contribution of local feedback mechanisms to the range of climate sensitivity in two GCM ensembles. Clim. Dynam.

[bib50] Wigley T.M.L, Richels L.R, Edmonds J.A (1996). Economic and environmental choices in the stabilisation of atmospheric CO_2_ concentrations. Nature.

[bib51] Woodward S, Roberts D.L, Betts R.A (2005). A simulation of the effect of climate-change induced desertification on mineral dust aerosol. Geophys. Res. Lett.

